# An anthropological approach to teach and evaluate cultural competence in medical students – the application of mini-ethnography in medical history taking

**DOI:** 10.3402/meo.v21.32561

**Published:** 2016-09-22

**Authors:** Jyh-Gang Hsieh, Mutsu Hsu, Ying-Wei Wang

**Affiliations:** 1 Department of Family Medicine, Hualien Tzu Chi Hospital, Hualien, Taiwan; 2 Department of Human Development and Psychology, Tzu Chi University, Hualien, Taiwan; 3 Department of Medical Humanities, School of Medicine, Tzu Chi University, Hualien, Taiwan

**Keywords:** mini-ethnography, cultural competence, holistic care, narratives

## Abstract

**Purpose:**

To use mini-ethnographies narrating patient illness to improve the cultural competence of the medical students.

**Methods:**

Between September 2013 and June 2015, all sixth-year medical students doing their internship at a medical center in eastern Taiwan were trained to write mini-ethnographies for one of the patients in their care. The mini-ethnographies were analyzed by authors with focus on the various aspects of cultural sensitivity and a holistic care approach.

**Results:**

Ninety-one students handed in mini-ethnographies, of whom 56 were male (61.5%) and 35 were female (38.5%). From the mini-ethnographies, three core aspects were derived: 1) the explanatory models and perceptions of illness, 2) culture and health care, and 3) society, resources, and health care. Based on the qualities of each aspect, nine secondary nodes were classified: expectations and attitude about illness/treatment, perceptions about their own prognosis in particular, knowledge and feelings regarding illness, cause of illness, choice of treatment method (including traditional medical treatments), prejudice and discrimination, influences of traditional culture and language, social support and resources, and inequality in health care.

**Conclusions:**

Mini-ethnography is an effective teaching method that can help students to develop cultural competence. It also serves as an effective instrument to assess the cultural competence of medical students.

To improve the quality of medical services, it is critically important that health care professionals must take into account the culture, language, health beliefs, and environmental differences found in patients (1). Thus, cultural competence is a crucial skill that helps health care providers to reduce inequality in health care (2–4). In 2000, the Liaison Committee on Medical Education established new standards requiring faculty and students to understand the manner in which people of diverse cultures and belief systems perceive health and illness and respond to various symptoms, diseases, and treatments (1, 5, 6). Similarly, the Accreditation Council for Graduate Medical Education outlined comprehensive cultural competency elements in the areas of patient care, professionalism, and interpersonal and communication skills (7). These standards make clear that cultural competence education must be integrated into training programs at every learner level (8). With these competencies, healthcare providers can cross the barriers of cultural differences, communicate successfully with patients, and achieve the goal of holistic care (9–11).

At present, questionnaires are the primary means of assessing cultural competence, which are self-report surveys of knowledge, attitude, or skills (3, 11–14). However, despite the efforts by researchers to increase the validity of the questionnaires, this approach is still subject to bias (10–12). Some researchers believe that self-assessment questionnaire respondents are extremely susceptible to social desirability effects and tend to choose answers that will create a good impression (12, 14). Teal et al. (15) and Thompson et al. (16) stated that respondents commonly overestimate their cultural competence. The practicality of these questionnaires has also been questioned on account of the fact that the questionnaires merely focus on the knowledge, attitude, and skills of the respondent and may oversimplify intercultural sensitivity and the deeper connotations of cultural influences (17, 18). Quantitative self-assessment questionnaires cannot accurately measure whether a student truly possesses adequate sensitivity and knowledge regarding cultural issues in a clinical setting. Qualitative research, whether standing alone or in a mixed method, adds rich information to any investigation otherwise not discovered through quantitative approaches (19). We have to use qualitative analysis to increase the breadth and depth of cultural competence assessments (14).

Ethnography, used in conjunction with medical anthropology, is an effective and qualitative research instrument that provides a complete understanding of the individuals residing in certain social environments, including their life, culture, actions, and unique views (20). Similar to anthropologists, clinical doctors must document the medical history of patients and their beliefs, feelings, and reactions in the face of disease and treatment in their medical records. A medical record is a narrative record and can be considered a mini-ethnography of the individual patient (21). They help doctors understand the illnesses and life experiences of the patient and also help doctors empathize with the patient. This experience marks the beginning of a medical student's entrance into the patient's world and enables an understanding of the causes and consequences of the illness so that they can establish corresponding treatment procedures, assess the degree of improvement in patient symptoms, and ease the suffering of the patient (22).

The application of ethnography to health care not only improves cultural competence but also enhances the application of evidence-based practice through an understanding of the actual living conditions of certain communities and the conceptualization of the meaning of their health behavior (23, 24). Nevertheless, the actual application of ethnography to medical education is comparatively rare, and past applications have simply featured ethnographic methods focused on medical student culture in universities and in workplace settings (19, 24, 25). One of the objectives of our curriculum was to use mini-ethnographies narrating patient diseases to improve the culture competence of the medical students. The students were required to write mini-ethnographies about their patients, analyze these records, and thereby understand what they lack in cultural competence.

## Method

The medical humanities and communication course started between September 2013 and June 2015, and the participants were sixth-year medical students doing their internship at a medical center in eastern Taiwan. During the first class of each semester, the students were trained to write mini-ethnographies and given reference principles and writing samples. The students were required to write a mini-ethnography, the assignment of the semester, for one of the patients in their care and hand it in at midterm. Then, in groups of three, the students discussed the contents of their assignments with their advising teacher, who gave them feedback and guided them in reflection in an effort to strengthen the effects of the mini-ethnographies on cross-cultural care training.

A written guide released for the assignment included an introduction to doctor-patient communication skills to help students to understand the experience and feelings of the patients regarding their diseases and illnesses. The eight questions proposed by Arthur Kleinman (26) regarding the cultural aspect of cross-cultural medical assessments served as the focus of the patient records: 1) What do you think has caused your problem? 2) Why do you think it started when it did? 3) What do you think your problem does inside your body? 4) How severe is your problem? Will it have a short or long course? 5) What kind of treatment do you think you should receive? 6) What are the most important results you hope to receive from this treatment? 7) What are the chief problems your illness has caused you? 8) What do you fear most about your illness/treatment? The students were encouraged to refer to these eight questions when constructing the framework for their records. For providing anonymity and clearly distinguishing different students’ articles, each student was assigned a specific code (such as B29, C16, and C5) to represent his identity during the process of data analysis. The mini-ethnographies were analyzed using NVivo 10 software (an instrument developed to help users organize and analyze non-numerical or unstructured data) with focus on the various aspects of cultural sensitivity and a holistic care approach.

## Results

During the 2 years of this study, 91 students handed in mini-ethnographies, of whom 56 were male (61.5%) and 35 were female (38.5%). From the mini-ethnographies, we derived three core aspects: 1) the explanatory models and perceptions of illness, 2) culture and health care, and 3) society, resources, and health care. Based on the qualities of each aspect, we derived nine secondary nodes: expectations and attitude about illness/treatment, perceptions about their own prognosis in particular, knowledge and feelings regarding illness, cause of illness, choice of treatment method (including traditional medical treatments), prejudice and discrimination, influences of traditional culture and language, social support and resources, and inequality in health care (Table 1).

**Table 1:** Cultural and holistic care issues students mentioned in their mini-ethnographies

Primary and secondary nodes	Number (proportion) of students mentioning the node	Number of mentions
Explanatory models and perceptions of illness		
Expectations and attitude about illness/treatment	55 (61.1%)	67
Perceptions about their own prognosis in particular	40 (44.5%)	46
Knowledge and feelings regarding illness	35 (38.9%)	43
Cause of illness	14 (15.6%)	16
Culture and healthcare		
Choice of treatment method – traditional medical treatment	23 (25.6%)	23
Prejudice and discrimination	5 (5.6%)	5
Influences of traditional culture and language	2 (2.2%)	4
Society, resources, and healthcare		
Social support and resources	27 (30.0%)	35
Inequality in health care	6 (6.7%)	11

### Explanatory models and perceptions of illness

Approximately 6 out of 10 students (55 students in total) attempted to understand what the expectations and attitudes about illness/treatment of the patients. Less than half (44.5 and 38.9%) of the students described what the patients knew and how they felt about their illnesses and what their perceptions of their own prognosis. In contrast, only 15.6% of the students cared about what their patients felt had caused their illnesses.

Student B29 remarked on how his patient attributed his disease to past life karma, which eased the misery caused by his cancer:

Even though the cancer has had a profound impact on his life, he accepts it and sees it as spiritual practice. He believes that the sins he committed in his previous life can only be forgiven after he completes this spiritual practice, in this life, and that the heavens must have arranged this because he has not repaid his debt. The fact that he has not died yet is because he has yet to serve this purpose.

This fatalism does exist not only in Buddhism but also in Christianity, which has a similar explanatory model in which suffering is a trial given by God. In addition to the influence of religious views on the explanatory models of illnesses, Student C5 described the influence of explanatory models on treatments, stating that patients who are overly fatalistic can allow their illnesses to get out of control:

She does not believe that her heart failure was exacerbated by her drinking or indiscriminate use of painkillers. Rather, she thinks that this is a punishment given by God, so she must pray with greater earnest. She tries to participate in all church events and possesses very little insight with regard to her own prognosis.

### Traditional medicine and health care

Students were less familiar with traditional medical treatments. Most students focus on western medicine and rarely see the need for traditional medical treatments when discussing how their patients choose treatment options. Only five presented their observations regarding prejudice and discrimination, and only two students mentioned the influences of traditional culture and language. Traditional culture, language, prejudice, and discrimination are important aspects of cultural sensitivity in healthcare work. This could potentially reveal that culture is still not a primary concern for the medical students.

Student C16 noticed his patient's desire for a traditional Chinese medicine (TCM) treatment. However, he questioned its effectiveness and even suggested his patient, Grandma Chen, not to administer TCM on her own.

Up to the time of her discharge, Grandma Chen was still thinking of the tonics and herb medicine that would benefit her liver and prevent her from needing surgery. I reminded her not to *administer* traditiona*l herb medicine on her own*.

Student B26 was one of the few to notice that differences between ethnic groups in language and traditional medical treatments led to inequality in health care:

Ms. Lin is Amis (an aboriginal tribe). Originally, their medicine focused on traditional folk remedies, or they relied on witch doctors. In her old age, she was forced to accept western medicine, which she had never encountered before. The terms *surgery* and *chemotherapy* may be as familiar to us as the alphabet we use every day, but for Ms. Lin, who had lived the majority of her life in traditional Amis culture, the shock was definitely no trivial matter.

### Society, resources, and health care

Society, resources, and health care also did not appear to be a point of focus for medical students. While 23% (27 students) noticed whether their patients had or lacked social support and resources, only 5% (6 students) were aware of the impact of healthcare inequality on the medical care and treatments of their patients.

Student C37 raised a very practical issue, stating that without continued care and concern, the distribution of resources is merely a temporary solution to economic problems:

In government social welfare institutions, the donations from good Samaritans may provide these children with the funds or resources that they need for living or medical treatments. However, what they lack even more is care and patience from others. After all, few people are willing to put much effort into taking care of someone who has no close relationship with them.

Student A17 described the unequal distribution of medical resources due to geological barriers, which made the residents of remote areas ‘orphans’ in medical care:

Due to the meager medical resources in Fuli Township (a remote township in Taiwan), the patient's husband is reluctant to have her discharged from the hospital. They are worried that they will not be able to get medical treatment in time if an emergency occurs after they return home.

## Discussion

The text analysis of the student assignments encompasses the primary aspects of cross-cultural care. The cultural aspect of illness interpretation as presented by Arthur Kleinman seems to apply to what the patients or their family believe to have caused the illness, their knowledge and feelings regarding the illness, their expectations and attitude about illness/treatment, influences of traditional culture and language, and the choice of treatment method all correspond directly to the eight questions. Another critical issue in students’ narrative writing can be classified as social determinants of health, including prejudice and discrimination, social support and resources, and inequality in health care. This is another aspect of cross-cultural health care (10, 27) that can be observed via text analysis.

Medical students are trained in modern western medicine, so they are accustomed to the explanatory models based on the framework of the biomedical model and naturally use these concepts to explain the problems of their patients (23, 26). However, this also causes them to overlook the importance of their patients’ explanatory models as well as the differences between their patients and themselves in terms of values. As a consequence, they forgo the chance to understand their patients further, which can influence the effectiveness of treatments (23, 28). What students must learn from this is that when patients have different views from their doctors regarding the occurrence and treatment of their illness, the patients may choose to believe their own judgment and doubt the judgments of their doctors. If the treatment is not as effective as was anticipated, they will be even more likely to blame it on their doctor and turn to other types of informal (unconventional) treatment (29, 30). It is therefore necessary for the students to understand the culture of their patients and the concepts of folk remedies in order to be aware that these concepts can influence the medical care-seeking behavior of the patients and adjust their thinking patterns, so that they can better understand their patients during doctor–patient communication (31, 32). Factors that appeared to influence the process by which medical students accommodated cultural needs included lack of experience in cross-cultural encounters and insufficient training in cultural knowledge and skills. Role modeling and personal reflections, ideally guided by faculty, are the most effective techniques that help to build upon students’ initial exploratory understanding of patients’ experiences (33).

Aside from the issues related to the explanatory models proposed by Arthur Kleinman, the mini-ethnographies written by the students also reveal issues with social determinants such as social support, resources, and inequality in health care. These are also integral matters when attempting to transcend racial and ethnic differences in cultural competence. Differences in race/ethnicity, socioeconomic status, place of residence, sexual orientation, or even age can mean that every encounter between doctor and patient is a cross-cultural interaction. This presents a gap that needs to be bridged in health care (10, 34, 35). Further effort should therefore be invested in cultural competence training courses for medical students (27).

Generally speaking, ethnographies represent the perspectives of the interviewees. However, the mini-ethnographies in this study were made to analyze the writers, that is, the medical students. By reflecting on the records of doctor–patient interactions and conversations, doctors can make decisions based on their patients’ beliefs and understand the interactions between their patients and the society in which they live. This is an ideal use of the biopsychosocial model (36). When the medical students conversed with the interviewees, they asked questions based on the reference guidelines provided in the course. By analyzing the mini-ethnographies, we can in turn examine the cultural sensitivity of the interviewers (the medical students). Of course, it is difficult to completely divulge the cultural sensitivity of the students based on the analysis of a single assignment, but from a methodological view, text analysis can quantify established items and units of analysis according to the text analysis rules and compare the number of times certain words and symbols appear, giving an objective perspective of the data being analyzed (37, 38).

## Conclusion

In teaching medical students’ cultural competence, we should teach them not only about ethnic and cultural differences but also how to examine and understand the explanatory models of their patients with regard to their illnesses. With proper training, medical professionals can apply more appropriate doctor–patient communication and develop treatments that comply with the life experiences and social contexts of the patients (10, 39). This requires clinical workers to have moderate familiarity with this framework, and mini-ethnography is an effective teaching method that can help students to develop cultural competence. It also serves as an effective instrument to assess the cultural competence of medical students.

## Conflict of interest and funding

The authors have not received any funding or benefits from industry or elsewhere to conduct this study.
